# HPV58 E7 Protein Expression Profile in Cervical Cancer and CIN with Immunohistochemistry

**DOI:** 10.7150/jca.50816

**Published:** 2021-01-18

**Authors:** Qiaoli Zheng, Xianzhen chen, Rui Han, Jiang Zhu, Hui Wang, Lingjing Chen, Yinjing Song, Luxia Chen, Hao Cheng, Na Jin

**Affiliations:** 1 Department of Dermatology, Sir Run Run Shaw Hospital, School of Medicine, Zhejiang University, Hangzhou, Zhejiang Province, China.; 2 Department of Pathology, The First People's Hospital of Fuyang, Hangzhou, Zhejiang Province, China.; 3 Department of Dermatology, Hangzhou Children's Hospital, Hangzhou, Zhejiang Province, China.

**Keywords:** cervical cancer, CIN, E7, HR-HPV, immunohistochemistry

## Abstract

**Background:** The persistent infection of high-risk human papillomavirus (HR-HPV) is one of the most common causes of cervical cancer worldwide, and HPV type 58 (HPV58) is the third most common HPV type in eastern Asia. The E7 oncoprotein is constitutively expressed in HPV58-associated cervical cancer cells and plays a key role during tumorigenesis. This study aimed to assess the HPV58 E7 protein expression in the tissues of cervical cancer and cervical intraepithelial neoplasia (CIN).

**Methods:** A total of 67 HPV58-positive cervical samples were collected, including 25 cervical cancer samples and 42 CIN samples. All the tissues were examined by HPV58 E7, p16^INK4a^ and Ki67 immunohistochemistry (IHC). At last, we analyzed their association with clinical and pathological variables.

**Results:** HPV58 E7 expression was detected in 96% of the HPV58 DNA-positive cervical cancer tissues and 85.7% of HPV58-positive CIN tissues. 65 samples of cervical cancer and CIN tissues had p16-positive staining, while 59 samples were Ki-67 positive.

**Conclusions:** HPV58 E7 protein is highly expressed in both cervical cancer and CIN tissues. HPV58 E7 IHC could be sensitive and specific for evaluating HPV-driven cervical cancer and pre-cancerous lesions, in combination with p16 and Ki-67 IHC.

## Introduction

Cervical cancer is one of the most common female genital cancers 1. High-risk human papillomavirus (HR-HPV) is one major cause of cervical cancer, which accounts for over 500,000 new cancer cases and over 250,000 cancer deaths per year 2. HPV type 16 (50%) and 18 (15%) are the most common subtypes worldwide. However, HPV type 58 (HPV58) plays a more prominent role in cervical cancer and pre-cancer (cervical intraepithelial neoplasia 1 (CIN1), CIN2, CIN3) in Asian countries. It is identified in 11.5% to 28% of cervical cancer patients in China 3.

HPVs are small, nonenveloped double-stranded DNA viruses that replicate their genomes in the nuclei of host cells (the keratinocytes). HR-HPV infection leads to the activation of viral gene expression, including the early genes *E6* and *E7* 4. Their expression is important for cellular transformation process. The E7 oncoproteins degrade and inactivate the retinoblastoma tumor suppressor protein pRB, contributing to the malignant transformation of the host cells 5, 6. Specifically, the genomic and transcriptomic profiles of HPV58 in cervical neoplasia have been studied, and variants such as *T20I* and *G63S* substitutions at *E7* of HPV58 were proposed to confer a significantly higher risk for cervical neoplasia 7, 8. However, the proteomes of HPV58, especially E7 oncoprotein, has not been well addressed. The detection of HPV58 E7 protein could be critical both in establishing the diagnosis of malignancy and assigning an HPV-related etiology to malignancy.

To date, a commercial antibody to HPV58 E7 for immunohistochemistry detection is still unavailable. We have produced and verified the specific polyclonal antibody against the HPV58 E7 protein 9. In this study, we aimed to detect the HPV58 E7 protein expression using this antibody in the tissues of HPV58-positive cervical cancer and CIN with IHC. Meanwhile, we detected the Ki-67 and p16 expression, analyzed the correlations between HPV58 E7, p16, or Ki-67 expression levels and clinical and pathological variables. The E7 oncoproteins expression would be a potential marker for HPV58-related cervical cancer and CIN diagnosis and disease progression.

## Methods

### Study population

This study was performed according to the Declaration of Helsinki. The study protocol was approved by the Ethics Committee of Sir Run Run Shaw Hospital of Zhejiang University School of Medicine, China.

The following inclusion criteria were required to be included in the study: (1) histologically confirmed diagnosis of cervical cancer or CIN; (2) positive result of the HPV58 DNA test. Exclusion criteria included previous treatment for the cervical disease (including loop electrosurgical excision procedure (LEEP), cold-knife conization, cryotherapy, LASER therapy, or hysterectomy, prior chemotherapy or radiation treatment for cervical neoplasia, pregnancy, HIV infection, previous or co-existence of other malignant diseases. 25 cervical cancer patients and 42 CIN patients who were HPV58-positive detected by polymerase chain reaction (PCR) were included between January 2009 and August 2016 from Sir Run Run Shaw Hospital of Zhejiang University School of Medicine. Written informed consent was obtained from all the participants. Formalin-fixed and paraffin-embedded (FFPE) specimens from cervical cancer patients and CIN patients diagnosed according to the WHO classification of tumors of female reproductive organs by the Division of Pathology and were collected by the Departments of Obstetrics and Gynecology, Sir Run Run Shaw Hospital. Authors could access information that could identify individual participants during and after data collection.

### Patient evaluation and disease monitoring

All patients' routine history had been taken, a physical examination, HPV test and cervical biopsy for morphological analysis. Morphologic diagnoses were based on consensus review by 2 subspecialty gynecologic pathologists. When morphology was equivocal, the diagnoses were adjudicated by a third gynecologic pathologist. LEEP or hysterectomy was performed and the removed tissues were transferred for histopathological examination. Besides, we collected the data on the degree of cell differentiation, tumor size and lymph node metastasis. Cervical carcinoma or CIN recurrences would be detectable by cervical biopsy during follow-up visits.

### HPV genotyping assay

Total DNA was extracted from 200 µl exfoliated cervical cell suspension with the HPV DNA extraction kit (TELLGEN, Shanghai, China) according to the manufacturer's instructions. Using the HPV DNA genotyping kit (TELLGEN, Shanghai, China), the Luminex® xMAP™ technology can quickly and quantitatively detect 27 types of HPV (high risk subtypes 16, 18, 31, 33, 35, 39, 45, 51, 52, 56 , 58, 59, 66, 68, 26, 53, 82, and low risk subtypes 6, 11, 40, 42, 43, 44, 55, 61, 81, 83). Briefly, 5 µl extracted DNA solution was used for PCR in a reaction (20 µl) containing 10 µl PCR mixture, 5 µl primer mixture, and 0.8 µl rTaq polymerase. The PCR amplification was performed and 3 µl products were added to microsphere hybrid for hybridization procedure on an ABI9700 (ThermoFisher, Waltham, U.S.A.). Each reaction pore was added 75 µl SA-PE. The mixture was detected on Luminex200 multifunctional flow detector (R&D system, Minneapolis, U.S.A.).

### Immunohistochemistry analysis

FFPE HPV58-positive (detected by PCR) cervical cancer or CIN sections (8-µm) were used for immunohistochemical staining of the HPV58-E7 protein 9, anti-p16 mouse monoclonal antibody (mAb) (1:500, GT201302, Gene, Shanghai, China) and anti-Ki-67 mouse mAb (1:500, MAB-0672, MXB, Fuzhou, China). Endogenous peroxidases in the de-paraffinized sections were quenched with 0.3% hydrogen peroxide in 60% methanol for 20 min. Non-specific adsorption was minimized by incubating the sections in 2% normal goat serum (Beyotime, Shanghai, China) in PBS for 20 min. The sections were then incubated with anti-HPV58-E7 rabbit polyclonal antibody (1:500) at room temperature for 2 hours. Then, the sections were washed with PBS and incubated with an HRP-conjugated goat anti-rabbit IgG secondary or goat anti-mouse antibody (1:1000, Beyotime, Shanghai, China). The immunoreactions were visualized by incubating the sections for 3 minutes in a 0.1% 3, 30-diaminobenzidine (DAB) solution and counterstained with hematoxylin for 8 mins. The stained cells were observed under a microscope (Olympus, Tokyo, Japan).

### Immunohistochemical data analysis

The staining intensities of HPV58 E7, Ki-67 and p16 were graded on a scale from 0 to 3+ (1+: weak, 2+: moderate, and 3+: strong) 10. In brief, the positive reaction for three antibodies was scored into four grades, according to the intensity of the staining: 0, 1, 2 and 3. The percentages of positive cells were also scored into five categories: 0 (0%), 1 (1-25%), 2 (26-50%), 3 (51-75%) and 4 (76-100%). The product of the intensity by percentage scores was used as the final score, which was categorized into four groups: 0 (<1), 1+ (1-3), 2+ (3-5), 3+ (>5). The immunostained specimens were independently evaluated by two blinded investigators. There was close agreement (>90%) between the two investigators; in the case of any disagreement, final grading was determined by consensus.

### Statistical analysis

Categorical data analysis was conducted using the χ^2^ test. Either the Student's t-test or Wilcoxon test was performed to determine the differences between groups. Spearman's correlation was used for the correlation analysis. Results were considered statistically significant at *p* < 0.05. All statistical analyses were done with SPSS22.0.

## Results

### Patient characteristics

Patient demographic and disease characteristics are summarized in Table 1. In total, 67 patients (25 cancer patients and 42 CIN patients) were included in the study. The median age at the time of diagnosis was 43 years old (range 27-65). Among 25 cervical cancer patients, 21 patients (84%) were in cervical cancer stage 1, 1 (4%) in stage 2, 2 (8%) in stage 3 and 1 (4%) in stage 4. Of 42 CIN patients, 4 (9.5%) were diagnosed as CIN1, 14 (33.3 %) were diagnosed as CIN2, and 24 (57.1%) were diagnosed as CIN3. The median follow-up time was 41 months (range 2-240). It showed two cases of relapse, and two cases of death.

### Immunohistochemical results

**Figure 1:**
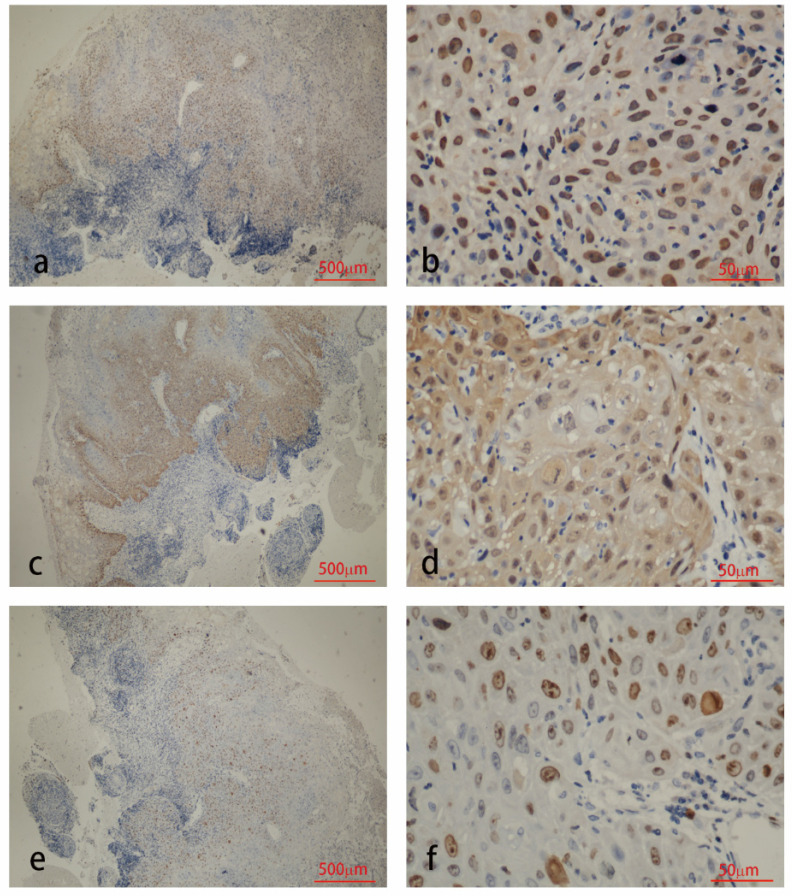
Immunohistochemistry staining for HPV58 E7 (a:×40, b:×400), p16 (c:×40, d:×400) and Ki67 (e:×40, f:×400) in cervical cancer biopsies.

**Figure 2:**
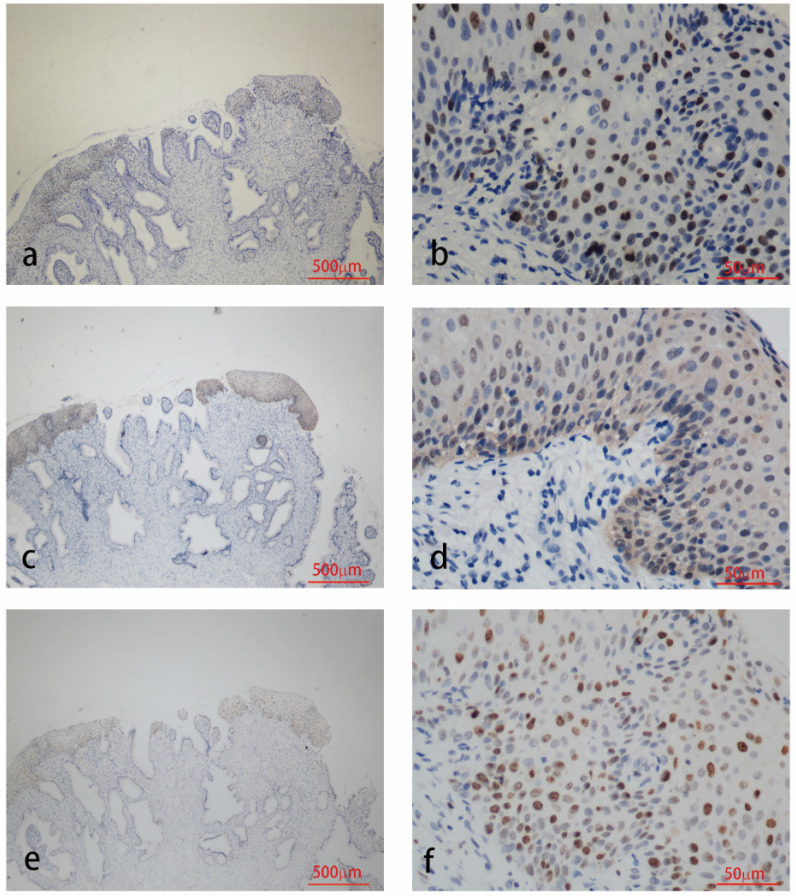
Immunohistochemistry staining for HPV58 E7 (a:×40, b:×400), p16 (c:×40, d:×400) and Ki67 (e:×40, f:×400) in CIN biopsies.

The results of immunohistochemical tests for HPV58 E7, p16 and Ki-67 are presented in Table 2. The expression of the HPV58 E7 protein was confirmed in 96% of the HPV58 positive cervical cancer tissues examined, and in 85.7% of HPV58 positive CIN tissues. The intensity of E7 staining was weak in 36% of cancer cases, moderate in 28% of cancer cases and strong in 32% of cancer cases. The intensity of E7 staining was weak in 42.9% of CIN cases, moderate in 23.8% of CIN cases and strong in 19.0% of CIN cases. P16 expression was found in 96% of the cancer group, and 97.6% of the CIN group. The intensity of p16 staining was weak in 4% of cancer cases, moderate in 4% of cancer cases and strong in 88% of cancer cases. The intensity of p16 staining was weak in 26.2% of CIN cases, moderate in 38.1% of CIN cases and strong in 33.3% of CIN cases. The expression of the Ki-67 protein was demonstrated in 96% cancer cases, and 83.3% CIN cases. The intensity of Ki-67 staining was weak in 0% of cancer cases, moderate in 28% of cancer cases and strong in 68% of cancer cases. The intensity of Ki-67 staining was weak in 7.1% of CIN cases, moderate in 54.3% of CIN cases and strong in 11.9% of CIN cases. As shown in Table 2, the expression level of HPV58 E7, p16, and Ki-67 in cervical cancer tissues were higher than in CIN tissues. Representative photographs of positive color reactions are shown in Figure 1 and Figure 2.

### Correlations between HPV58 E7, p16, or Ki-67 expression levels and clinical and pathological variables

Base on Table 2, we subdivided the staining results into 2 groups for further analyses: group 1 (weak and moderate staining), group 2 (strong staining). When we analyzed the age distribution of cervical cancer patients (Table 3), we found that HPV58 E7 expression level increased and Ki-67 expression decreased in older women (Table 4, *p*<0.05). Correlations of HPV58 E7, p16 and Ki-67 expression levels with clinical and histological variables in cervical cancer patients were demonstrated in Table 4. There were no significant differences in clinicopathological factors, including the degree of differentiation, the diameter of tumor lesions, staging of the tumor, lymph node metastasis, relapse, age of menarche, age of marriage, times of fetation and times of abortion between patients with low-moderate and strong expression level of HPV58 E7, p16, and Ki-67. However, p16 expression was associated with HPV58 E7 and Ki-67 expression (Table 4, *p*<0.05). As revealed by Spearman's correlation, there's a positive correlation between p16 expression and HPV58 E7 (r=0.391, *p*=0.027) or Ki-67 expression (r=0.370, *p*=0.034). In CIN patients, there were no significant differences in clinicopathological factors between group 1 and group 2.

## Discussion

In this study, we demonstrated the HPV58 E7 oncoprotein expression pattern in cervical cancer and CIN with immunohistochemistry. We determined the association of HPV58 E7 with other clinical and molecular parameters in cervical cancer samples.

Previous studies demonstrated that HR-HPV infection is associated with a broad spectrum of carcinogenesis in a variety of anatomic locations including the uterine cervix, vulva, anus, penis, and head and neck 11, 12. The HPV 16, 18 types are mostly associated with cervical carcinoma worldwide 13. HPV-58 is a common subtype of HR-HPV, which accounts for a notable proportion of the cervical cancers in Asian countries. It has been identified in 11.5 to 28% of cervical carcinoma patients in China, 8% in Japan and 16% in Korea 14. HPV16, 58, and 52 were the three most dominant HPV genotypes in cervical intraepithelial lesions in Shanghai 15. In another study, they demonstrated HPV58 is the third most common HPV type in Eastern Asia overall 16.

DNA-based PCR is widely used in analyzing clinical specimens for the presence of HPV infection. However, the detection of DNA only indicates the presence of HPV and the expression of oncogenes are associated with progression to invasive cancer 17. The *E6* and *E7* genes are the main oncogenes of HR-HPVs 18. There should be an overexpression of the *E7* gene from the integrated HPV genome. Some studies demonstrated the presence of E7-specific antibodies in sera in connection with increased risk of cervical cancer that may be detected up to 5 years prior to diagnosis 19. However, the E7 antibodies were detected infrequently which might result from the low level of the HPV E7 antibodies in human sera. Another study showed seroprevalence of HPV18 E7 antibodies were significantly higher in HPV18 DNA-positive cervical cancer cases than controls. However, apparent limitations were observed. Firstly, cross-reactivity was evident among closely related HPV types. Secondly, one-third of cervical cancer cases showed no detectable E6 or E7 antibodies in sera among cases positive for HPV DNA 20. Thus, we detected the E7 protein level in tissues of cervical cancer and CIN patients with the self-prepared polyclonal antibody 9. The results revealed HPV58 E7 expressed in 96% of the HPV58 positive cervical cancer tissues examined, in 85.7% of HPV58 positive CIN tissues. The positive rate of E7 immunohistochemistry is relatively high in cervical cancer and CIN tissues. The method is worthy of clinical practice. The protein expression might be a useful marker for disease progression. However, there are still no type-specific commercial antibodies targeting HPV58 E7 oncoprotein in clinical application. In the current study, we evaluated the HPV58 E7 protein expression levels in cervical cancer and CIN samples by immunohistochemistry using a specific polyclonal antibody produced and verified by our research group 9. We found that HPV58 E7 protein is highly expressed in cervical cancer and CIN samples by immunohistochemistry using this antibody, which provides evidence to support the reliability and potential of this antibody in clinical usage.

The HPV E7 protein in cooperation with E6 is essential for oncogenic cellular immortalization and transformation. E6 and E7 respectively induce the degradation of tumor suppressors p53 and retinoblastoma protein pRb 18, 21. In addition, HPV E7 proteins associate with the pocket proteins, p107 and p130, via the Leu-X-Cys-X-Glu (LXCXE) motif in their conserved region (CR) 2 domain 22. The pocket proteins regulate G1/S transition by modulating the transcriptional activities of E2F transcription factors 23. E2Fs also have effects on many other cellular processes relevant to tumorigenesis, including cellular differentiation, apoptosis, and genomic instability 24. E7 proteins also interact with other proteins involved in cell proliferation, such as histone deacetylases 4, p21 and p27 cyclin-dependent kinase inhibitors 25 and components of the AP1 complex 26. A report has indicated that HPV DNA replicates randomly or once per cell cycle in basal cells. The possible mechanism has been proposed, it may depend on the levels of the viral E1 replication protein and the cellular DNA replication protein components such as mini-chromosome-maintenance proteins (MCMs) 27. A study showed HPV16-E7 transgenic keratinocytes with distinct cell surface markers attracted new antigen-presenting cell (APC) subsets to the epidermis. Transport of antigen to the draining lymph node by these APCs was markedly enhanced while antigen-processing was significantly impaired. They suggested E7 oncoprotein affected the phenotype and function of APCs and contributed to persisting infection with HPV 28. HPV E7 may also induce the upregulation of RRM2 and promote ROS-ERK1/2-HIF-1α-VEGF-induced angiogenesis 29. Furthermore, recent researches focused on immunotherapeutic approaches against E7-derived antigens in patients with cervical and oropharyngeal cancers 30, 31. A study found that E6/E7 mRNA expression level was higher in women with high grade squamous intraepithelial lesion (HSIL) and CIN grade 2 or higher 32. The combination of HPV-positive DNA and E6 or E7 serology could predict overall survival in individuals with HPV infections 33. We found that CIN patients showed a lower rate of HPV58 E7 IHC positive compared to malignancy patients. The detection of the HPV E7 protein allows the monitoring of the oncogenic activity of the virus.

The Ki-67 protein is a proliferation marker that is detected in the cell nuclei during the G1, S and G2 phases of the cell cycle and in mitosis. It has been elucidated that the protein is essential for the proliferation process 34. The p16^INK4a^, a cyclin-dependent kinase (CDK) inhibitor, decelerates the cell cycle by inactivating the CDKs that phosphorylates the retinoblastoma protein (RB) 35. The expression of E6 and E7 proteins leads to p16 upregulation through the RB-p16 pathway. Thus, p16 could be a useful biomarker to improve the diagnosis of cervical cancer and CIN in combining with conventional morphology 36, 37. The rates for p16 and Ki-67 expression correlate with the severity of cervical neoplasia 38-40. In this study, p16 expression was found in 96% of the cancer group, and in 97.6% of the CIN group. The expression of the Ki-67 protein was demonstrated in 96% cancer cases, and in 83.3% CIN cases.

There were some limitations in our study. First, a smaller sample will give a result that may not be sufficiently powered to extrapolate the statistical difference. Second, as most of the cases were early-stage cervical cancers, survival analysis couldn't be conducted.

## Conclusions

In conclusion, IHC for HPV58 E7 is a test with high specificity and sensitivity. The HPV58 E7 protein is highly expressed in cervical cancer and CIN patients. A novel marker for cervical cancer and CIN diagnosis and disease progression allows further implications for disease monitor. Larger studies are warranted to confirm our findings and to clarify the relationship between HPV58 E7 expression with clinical and pathological variables, which exhibits close correlations with other clinical and molecular parameters in cervical neoplasia, including p16 and Ki-67 expression. These findings provide evidence to support the reliability and potential of this antibody in clinical application 9.

## Figures and Tables

**Table 1:** Summary of patient demographic and disease characteristics in 67 patients

Characteristics	No. of pts
Gender (female)	67
Age	43 (27-72)
Follow-up duration (months, median, range)	41 (2-240)
Staging of tumor*	
Pre-cancer	
Stage I	42
Stage 2	21
Stage 3	1
Stage 4	2
**Differentiation**	1
Low	16
Intermediate	8
High	1
Unknown	42
**Lymph node metastasis**	
Yes	3
No	64
**Diameter of tumor**	
<1 cm	57
1-3 cm	5
>3cm	5
**Relapse**	
Yes	2
No	65

*International Federation of Gynecology and Obstetrics (FIGO) staging of carcinoma of the cervix uteri (2018).

**Table 2:** Immunohistochemical staining for HPV58 E7, p16, and Ki-67 in cervical cancer and CIN cases

	Group	Total cases (n)	HPV58 E7 expression level, n (%)				P16 expression level, n (%)				Ki-67 expression level, n (%)			
			0	1+	2+	3+	0	1+	2+	3+	0	1+	2+	3+
Cervical cancer	Stage 1	21	1 (4.8)	6 (28.6)	7 (33.3)	7 (33.3)	1 (4.8)	1 (4.8)	1 (4.8)	18 (85.7)	2 (9.5)	0 (0.0)	5 (23.8)	14 (66.7)
	Stage 2	1	0 (0.0)	1 (100.0)	0 (0.0)	0 (0.0)	0 (0.0)	0 (0.0)	0 (0.0)	1 (100.0)	0 (0.0)	0 (0.0)	1 (0.0)	0 (0.0)
	Stage 3	2	0 (0.0)	2 (100.0)	0 (0.0)	0 (0.0)	0 (0.0)	0 (0.0)	0 (0.0)	2 (100.0)	0 (0.0)	0 (0.0)	0 (0.0)	2 (100.0)
	Stage 4	1	0 (0.0)	0 (0.0)	0 (0.0)	1 (100.0)	0 (0.0)	0 (0.0)	0 (0.0)	1 (100.0)	0 (0.0)	0 (0.0)	0 (0.0)	1 (100.0)
	Total	25	1 (4.0)	9 (36.0)	7 (28.0)	8 (32.0)	1 (4.0)	1 (4.0)	1 (4.0)	22 (88.0)	1 (4.0)	0 (0.0)	7 (28.0)	17 (68.0)
CIN	CIN1	4	0 (0.0)	2 (50.0)	1 (25.0)	1 (25.0)	1 (25.0)	1 (25.0)	1 (25.0)	1 (25.0)	1 (25.0)	0 (0.0)	3 (75.0)	0 (0.0)
	CIN 2	14	0 (0.0)	5 (35.7)	4 (28.6)	5 (35.7)	0 (0.0)	2 (14.3)	5 (35.7)	7 (50.0)	3 (21.4)	1 (7.1)	8 (57.1)	2 (14.3)
	CIN 3	24	6 (25.0)	11 (45.8)	5 (20.8)	2 (8.3)	0 (0.0)	8 (33.3)	10 (41.7)	6 (25.0)	3 (12.5)	2 (8.3)	16 (66.7)	3 (12.5)
	Total	42	6 (14.3)	18 (42.9)	10 (23.8)	8 (19.0)	1 (2.4)	11 (26.2)	16 (38.1)	14 (33.3)	7 (16.7)	3 (7.1)	27 (64.3)	5 (11.9)

**Table 3:** Association between age and HPV58 E7, p16 and Ki-67 staining

	Age (n)					
	20-29 yrs	30-39 yrs	40-49 yrs	50-59 yrs	>60 years	Total
Cervical cancer	1	5	7	7	5	25
HPV58 E7 1+/2+	1	5	4	4	2	16
HPV58 E7 3+	0	0	3	3	2	8
P16 1+/2+	0	0	0	1	1	2
P16 3+	1	5	7	5	4	22
Ki-67 1+/2+	0	1	1	2	3	7
Ki-67 3+	1	4	6	5	1	17

**Table 4:** Clinical and histological correlations of immunohistochemical findings in cervical cancer (*p*-value)

	HPV58 E7	P16	Ki67	Diameter of tumor	Staging of tumor	Lymph node metastasis	Relapse	Differentiation	Age	Age of menarche	Age of marriage	Times of fetation	Times of abortion
HPV58E7	-	0.101	0.647	1.000	0.618	1.000	0.304	1.000	0.037*	0.426	0.629	0.582	1.000
P16	0.025*	-	0.025*	0.670	0.858	1.000	0.170	1.000	0.140	0.415	0.894	1.000	0.293
Ki-67	0.647	0.507	-	1.000	0.552	0.530	0.304	1.000	0.034*	0.635	0.470	0.098	0.108

*p<0.05.

## Abbreviations

HR-HPV: high-risk human papillomavirus
CIN: cervical intraepithelial neoplasia
IHC: immunochemistry
FFPE: Formalin-fixed and paraffin-embedded
LEEP: Loop Electrosurgical Excision Procedure
PCR: polymerase chain reaction
DAB: diaminobenzidine
LXCX: E Leu-X-Cys-X-Glu
CR: conserved region
MCMs: mini-chromosome-maintenance proteins
APC: antigen-presenting cell
HSIL: high grade squamous intraepithelial lesion
CDK: cyclin-dependent kinase
RB: retinoblastoma protein
FIGO: International Federation of Gynecology and Obstetrics
